# Complications in septoplasty based on a large group of 5639 patients

**DOI:** 10.1007/s00405-018-4990-8

**Published:** 2018-05-16

**Authors:** Justyna Dąbrowska-Bień, Piotr Henryk Skarżyński, Iwonna Gwizdalska, Katarzyna Łazęcka, Henryk Skarżyński

**Affiliations:** 1 Institute of Physiology and Pathology of Hearing, Warsaw, Poland; 2 World Hearing Center, Nadarzyn, Poland; 3 Department of Heart Failure and Cardiac Rehabilitation, 2nd Faculty, Medical University of Warsaw, Warsaw, Poland; 4 Institute of Sensory Organs, Kajetany, Poland

**Keywords:** Hematoma, Intraoperative complications, Nasal cartilages, Nasal septal perforation, Nose deformities

## Abstract

**Purpose:**

Septoplasty is a common surgical procedure used for correction of the nasal obstruction caused by a deviated septum. The aim of the study was to identify complications in septoplasty and analyze incidence depending on the surgical technique, based on the material from 2009 till 2017.

**Methods:**

The material consisted of 5639 medical records from patients aged 16–69, operated in the tertiary referral center. Patients were divided into two groups (2784 exclusively with septoplasty and 2855 with combined septoplasty and turbinoplasty). *Z* test for the equality of two proportions was made to investigate the assumption that the proportions from two populations are equal, based on two samples, one from each population.

**Results:**

Complications were listed according to international standards. Among the whole study group, different types of complications were noted in 193 patients (3.42%). The most frequent complication was excessive bleeding. Significant differences were observed between the two investigated groups. In patients with combined septoplasty and turbinoplasty septal hematoma, hyposmia, prolonged healing due to infection, adhesions and temporary reduced visual acuity were significantly more often encountered.

**Conclusions:**

Meticulous attention to detail in identifying the appropriate anatomy and maintaining good visualization is the key to a safe and effective septoplasty, enabling for very low complication rate.

## Introduction

Nasal airway obstruction is one of the most frequent presenting complaints reported to the otolaryngologist. Deviations of the nasal septum are extremely common. Anatomic studies on human skulls revealed that 75–80% of all humans have a septal deviation to some degree [1, 2]. Hence, that septoplasty is one of the most frequently performed operation by otolaryngologists through the world [3–7]. Aesthetic indications (e.g. deviated dorsum) are not as common as functional [6]. The most important area for the airway is the nasal vestibule and the valve region. From the functional point of view, correction of caudal part of the nasal septum is crucial.

The goal of the surgery is the creation of a caudal septal plate in triangular area between the so called K-area (fusion of septal cartilage, upper lateral cartilages, nasal bones and perpendicular plate at the bony-cartilaginous junction of the nasal dorsum). This plate should be straight, stable, of adequate size and fixed. Septal surgery has evolved over time from the traditional submucous resection to more reconstructive procedure. At the same time turbinate surgery continues to move in the same direction with an emphasis on mucosal and structural preservation.

An undesirable result of the septoplasty can be caused by a complication or by a mistake [8]. A complication is a misfortune that cannot be influenced by a surgeon. A mistake is the result of inadequate planning or execution of the surgery or follow-up period. If an undesirable result of the septoplasty happens to a specialist, it should be considered whether it was a complication or a mistake. Unfortunately, such a precise distinction is in many cases impossible. Therefore, thorough analysis of the anatomic conditions and prevention of the potential undesirable agents are crucial aspects of the operation. Potential complications after septoplasty and turbinoplasty according to the current literature are listed in Table 1.

**Table 1:** Potential complications of septoplasty and turbinoplasty

Potential complications of septoplasty	Potential complications of turbinoplasty
Hemorrhage/septal hematoma	Hemorrhage
Septal perforation	Infection
Adhesions	Atrophic rhinitis
Adhesions	
Structural deformity (saddle nose, nasal tip ptosis, angulation of the nasal dorsum)	
Anosmia/hyposmia	
Infection/septal abscess/toxic shock syndrome	
Tooth anesthesia	
Endocranial complications (CSF leak, pneumocephalon)	
Ocular complications	
Cardiac/medical complications	

The aim of this study is to assess and compare complications after septoplasty and septoplasty with turbinate reduction. In the literature there is a lack of studies on such a large population. We also feel there is a strong need to discuss with patients complications based on a large group of patients while obtaining informed consent.

## Materials and methods

### Patients

The study was conducted from 01.2009 to 12.2016. In 5639 patients septal surgery was carried out in our tertiary referral center; in 2855 cases septoplasty was combined with lower turbinate surgery. Patients in whom the endoscopic septoplasty, septorhinoplasty and septoplasty with sinus surgery was done, were excluded from this study. The mean age of patients from the septoplasty group was *M* = 36.08; SD = 12.45 years (range 17–68). In the septoplasty and turbinoplasty group, the mean age of patients was *M* = 35.25; SD = 11.61 years (range 16–69). In both groups there was a predominance of male patients (65% men and 35% women).

The complications were gathered retrospectively using patients’ medical records and entered into the database, where the source data were entered such as patients initials, age, gender, operation time, operation type and observed complications (mentioned by the patient and also asked for by the physician). The average follow up after surgery was 12–34 months. 3.68% of the patients were lost during the follow-up, meaning they were present only once post-op for splints removal. Each examination included anterior rhinoscopy, endoscopic evaluation of septum, lateral nasal wall and turbinates from nostril till nasopharynx. We obtained a written informed consent from a one representative patient with a large septal perforation to use his face picture in the publication.

### Surgery

Upon surgery, a hemitransfixion incision was typically utilized to open the caudal septum, but some variations starting more posterior could be also appropriate. We must emphasize that every septum is unique, thus the surgery should be tailored and adapted to the individual patient [9]. Typically, there were five steps during septoplasty:

1. Exposure of the divided septum.
2. Release of the forces that cause angulations.
3. Realignment of the septum.
4. Reimplantation of the crushed cartilage.
5. Closure with absorbable sutures and splints placed in each side of the nasal cavity for 7 days and packing for 24 h. There were five surgeons performing this type of procedure.

The turbinate reduction was typically performed using 4 MHz radiofrequency generator Curis made by Sutter Medical.

Twenty-four hours after surgery, we took out the packing and the patient was under observation for another 24 h to ensure there was no bleeding, before he was discharged.

The post op indications were oral antibiotics for 7 days, nasal rinses 4–6 times daily, mupirocin 3× daily for 14 days.

### Statistical analysis

The frequency of different types of complications was analyzed and compared for septoplasty versus septoplasty with turbinoplasty. The *Z* test for the equality of two proportions was made to investigate the assumption that the proportions from two populations are equal, based on two samples, one from each population [10]. *p* values lower than 0.05 were accepted as significant. The IBM SPSS software version 24 was used for statistical analysis.

## Results

**Fig. 1:**
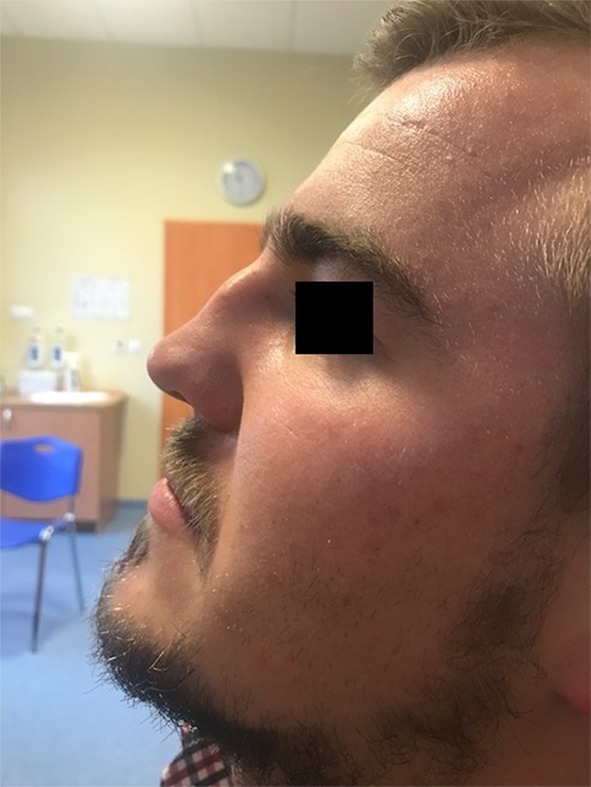
Collapse of the middle third of the nose after septoplasty in a male patient

**Fig. 2:**
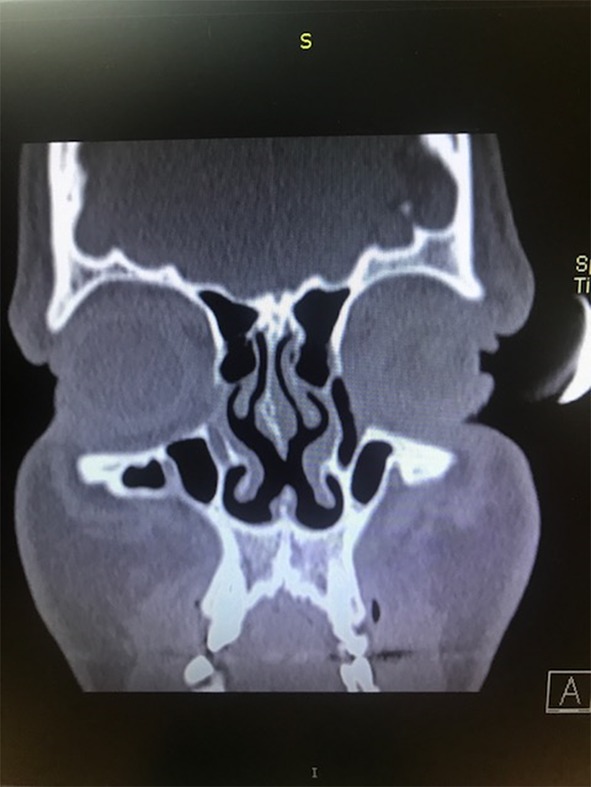
The computed tomography scan of the septal perforation in the same patient

**Fig. 3:**
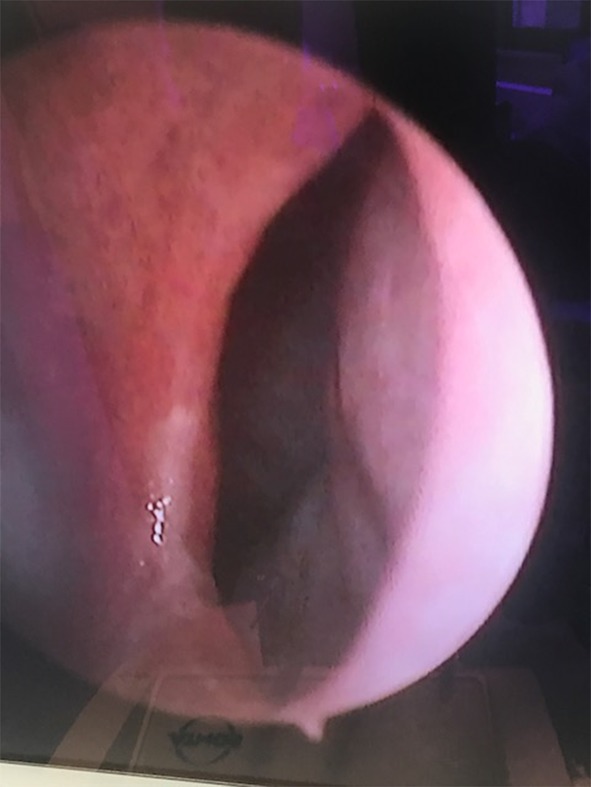
The septal perforation in the same patient in the endoscopic view

Among the whole study group, different types of complications were noted in 193 patients (3.42%). Some of the patients developed more than one complication and that is why the overall complication rate is lower than the sum of all complications incidence. The most frequent complication was excessive bleeding (3.3%) (Table 2), which required additional packing with absorbable hemostatic mesh (e.g. Surgicel). In the case of substantial bleeding, however, splint removal was sometimes necessary to manage the bleeding site directly either with cautery or packing. If the bleeding was unnoticed or it was between the splints it could end up as a septal haematoma, which usually needed splint removal and revision in the operating room. Septal perforation was observed in 2.3% of the patients undergoing surgery. An example of such a complication is presented in Figs. 1, 2 and 3. Hyposmia (evaluated subjectively) was more often observed if turbinoplasty was also made, and resolved over 2–6 months post-op.

**Table 2:** Overall complication rate in our material

Complication	Septoplasty surgery (*N* = 5639)	
	*n*	%
Excessive bleeding	188	3.3
Septal perforation	131	2.3
Hyposmia (> 1 week < 6months)	176	3.1
Infection (prolonged healing)	178	3.1
Adhesions	21	0.3
Tooth/upper lip anesthesia	11	0.1
Ocular complications (temporary reduced visual acuity)	5	0.08

Infection or prolonged healing (dryness or excess of secretion, congestion, crusting for more than 1 week) was observed in 3.1% of patients. Full recovery after septal surgery was among 7–16 days, whereas after septal and turbinate surgery it was 22–43 days. Adhesions were very seldom, because we routinely used splints for 7 days after septoplasty, but in the group with concomitant turbinoplasty they were observed in 0.6% of cases.

Complication rates according to the type of surgery are presented in Table 3. Statistically significant, higher rate of hemorrhage, hyposmia, infection, adhesions and ocular complications was observed when septoplasty was combined with turbinoplasty. We observed crusting and dryness or secretions in the middle and posterior third of the lower turbinates for up to 6 weeks after the surgery, whereas after septoplasty mostly after splints removal the nose became patent, and patient had no more disturbances with nasal obstruction. Few patients reported the numbness around the upper lip or tooth, this was also temporary and probably was due to the fixation of the splints or septum to the anterior nasal spine. The least frequent complications were the ocular complications in the form of transient blurred vision. We have seen external nose deformities after septoplasty in less than 1%, but it is hard to certainly say if it was a complication, as there was a noticeable tip or nostril asymmetry or subluxation of the septum before the operation.

**Table 3:** Complication rate in septoplasty and septoplasty with turbinoplasty group

Complication	Septoplasty (*N* = 2784)		Septoplasty + turbinoplasty (*N* = 2855)		
	*n*	%	*n*	%	*p*
Excessive bleeding	72	2.6	116	4.1	0.0018
Septal perforation	56	2.0	75	2.6	0.1332
Hyposmia (< 6 months > 1 week)	21	0.8	155	5.4	0.0000
Infection (prolonged healing)	57	2.0	121	4.2	0.0000
Adhesions	4	0.1	17	0.6	0.0016
Tooth/upper lip anesthesia	7	0.3	4	0.1	0.0918
Ocular complications (temporary reduced visual acuity)	2	0.07	3	0.1	0.0003

## Discussion

Excessive bleeding was most frequent complication in our study, what is similar to other studies [7, 11]. There is one report of lethal bleeding from the internal carotid artery after fracturing the vomer by force [12].

Septal perforations, second most common complication in our study, may not be readily apparent at first follow up, or are asymptomatic so are probably underreported overall [5]. The rate of nasal septal perforation after septoplasty ranges from 1.6 to 6.7% [6, 7, 11]. Most commonly, perforations result from traumatic elevation of the mucosal flaps with opposing tears in the flap on either side. Careful and meticulous elevation of the mucosal flaps is the key to prevention as is making an effort to close any mucosal tears at the end of surgery. Small perforations with well-mucosalized edges may not require any suturing at all, and may not have any impact on the nasal airway per se. Yet another reason for septal perforation involves the placement of quilting sutures or sutures to maintain septal splint placement. If the suture is tight enough to cause ischemia and necrosis of the surrounding area, a perforation may result; we have seen it twice. In some patients, local mucosal trauma caused by intranasal steroid and nasal saline sprays is enough to cause a perforation. Another reason they could be caused is by healing complications due to infection, typically *Staphylococcus aureus*. We prevent this by advising our patients application of antibiotic ointment (Mupirocin) and irrigation with a saline solution 4–6 times daily to keep the splints clear and minimize potential crusting in post-op period. Surgical repair of a septal perforation, if indicated or desired, is delayed well beyond the initial healing period (6–12 months at a minimum).

Adhesions or spikes mostly occur between septum and inferior turbinate if a tear in the septal mucosa is opposite to a mucosal defect of the turbinate, especially after simultaneous interventions at the turbinate. Septal splints can prevent the formation of the adhesions. We observed this complication more often in allergic rhinitis patients; adhesions were not observed as long as the splints were in place, but usually were noticed on the second or third post-op visit.

Although turbinate reduction was done with RF, which is known to be less traumatic than laser ablation, we believe that prolonged healing, adhesions and hyposmia, more often observed, were due to impaired mucociliary function.

Infections after septoplasty and/or turbinoplasty are rare complications. The rate of local infection and septal abscess after septoplasty ranges from 0.4 to 12.0% [7]. Other infectious complications such as meningitis, brain abscess, cavernous sinus thrombosis [13] and endocarditis [14] are extremely rare. Transient bacteremia, however, is not infrequent. Bacteria were found in venous blood samples obtained immediately after the operation in 15% and after removal of packing even in 16.9% [15]. This may explain why some patients have a body temperature increase postoperatively without evidence of local infection. This raises the question of whether prophylactic antibiotics are mandatory. In a survey, 66% of the surgeons used antibiotics routinely after septoplasty because of the fear of postoperative infection [8]. From the authors’ point of view, prophylactic antibiotics are necessary as long as the splints are kept (usually 7 days post-op). There are case reports of toxic shock syndrome (TSS) after use of nasal packing and intranasal splints, the estimated rate is 0.0165%.

Changes in the external appearance of the nose are nowadays uncommon, but can occur [8, 16]. We present such a complication (Fig. 1), but the patient was operated elsewhere. The overall rate of significant change in the cosmetic appearance of the nose after septoplasty has been reported between 0.4–3.4% [7, 8]. However, some surgeons [17] suggest that this is underestimated complication and aesthetic changes have been noted in up to 21% of the patients. Often this is as a result of inadequate fixation of the septal cartilage when it has been mobilized leading to its posterior inferior rotation and slight settling of the dorsal septum reflecting a loss of tip support and sometimes collumellar retraction [18, 19]. This usually has more a cosmetic than functional implication and can be corrected if the patient is bothered by it, with cartilaginous dorsal onlay grafting through an endonasal approach.

Anosmia or hyposmia is most often temporary and is due to edema. Permanent anosmia resulting from nasal surgery, as reported in the literature [20, 21] is uncommon (0.3–2.9%). Persistent symptomatic septal deviation (nasal obstruction) may be apparent early or may present later. The surgeon should reexamine the nasal airway with particular attention paid to the nasal valve areas. Revision surgery, if needed, should be done by a surgeon well experienced in such cases and typically not before 6 months after the initial surgery.

Cerebrospinal fluid (CSF) rhinorrhea after septoplasty is a rare, but serious complication [22]. Prevention is most important with care when resecting high deviations. Limited studies have been done to investigate the rate of dental anesthesia of the upper incisors after septoplasty surgery, but it is a potential complication [11]. Ocular complication, including blindness, are very rare, but do occur [23, 24]. The theory for complete visual loss after septoplasty is that when epinephrine is injected under pressure into the mucosa of the septum or tissue surrounding the inferior turbinate there is a risk of a retrograde flow through the anterior ethmoidal artery into the ophthalmic artery, which can cause vasospasm of the end arteries to the optic nerve and retina. This hypo perfusion can induce optic nerve neuropathy and unfortunately there is no treatment available in the late stages with corticosteroids and vasodilators.

Atrophic rhinitis has been attributed to overly aggressive turbinate resection with chronic symptoms of nasal crusting, mucosal atrophy and nasal congestion. Empty nose syndrome is a similar condition more specifically referring to the symptoms of paradoxical nasal congestion. Despite many possible complications we should encourage our patients to undergo septoplasty. Recent studies show significant improvement in disease-specific quality of life, high patient satisfaction and decreased medication use [25].

There was a group of patients “not satisfied” with the surgery, meaning they did not notice any difference post operatively. When thinking about contraindications, we should also think about patients whose goals and expectations regarding surgical outcomes are completely discordant with the measurable objective of the surgical procedure (e.g., those seeking resolution of postnasal drip, cure of headache in the absence of any symptomatic nasal obstruction, or elimination of chronic cough) so they are not good candidates for surgery.

In conclusion, most frequent complications after septoplasty were excessive bleeding. More severe complications like hyposmia or inadequate vision were very rare and transient. Meticulous attention to detail in identifying the appropriate anatomy and maintaining good visualization is the key to a safe and effective septoplasty, enabling for very low complication rate. Commitment to proper postoperative care must be stressed to the patient and is crucial for the healing process.
